# Blocking Type I Interferon Production: A New Therapeutic Option to Reduce the HIV-1-Induced Immune Activation

**DOI:** 10.1155/2012/534929

**Published:** 2011-11-29

**Authors:** Moritz Ries, Kathrin Pritschet, Barbara Schmidt

**Affiliations:** Institute of Clinical and Molecular Virology, German National Reference Centre for Retroviruses, Friedrich-Alexander-Universität Erlangen-Nürnberg, Schlossgarten 4, 91054 Erlangen, Germany

## Abstract

Highly active antiretroviral therapy has dramatically improved the morbidity and mortality of HIV-1-infected individuals. A total of 25 licensed drugs provide the basis for an optimized virus-suppressive treatment of nearly each subject. The promises of immune reconstitution and normal life expectancy, however, fall short for a number of patients, either through inadequate recovery of CD4+ T-cell counts or the occurrence of non-AIDS defining malignancies. In this respect, the prevalence of Epstein-Barr virus-associated Hodgkin lymphoma and human papillomavirus-related anal neoplasia is rising in aging HIV-1-infected individuals despite antiretroviral therapy. An important cause appears to be the HIV-1-induced chronic immune activation, propagated by inappropriate release of proinflammatory cytokines and type I interferons. This immune dysregulation can be reduced *in vitro* by inhibitors blocking the endosomal acidification. Recent data suggest that this concept is also of relevance *in vivo*, which opens the door for adjuvant immunomodulatory therapies in HIV-1 infection.

## 1. Old Problems Solved: New Arise

In the past 15 years, the antiretroviral therapy has considerably advanced. Due to efforts of basic and clinical sciences and pharmaceutical companies, a total of 25 antiretroviral drugs are available which target different steps of the HIV-1 replication cycle, namely, entry, reverse transcription, integration, and processing of the gag-pol precursor [1]. This variety of drugs allows to select the optimal antiretroviral combination for nearly each HIV-1-infected individual [2], promising an efficient and long-term virus suppression with reconstitution of the immune system and almost normal life expectancy. With growing experience in antiretroviral treatment, however, evidence also increases that not all HIV-1-infected patients profit or continue to profit from the blessings of antiretroviral therapy. Obviously, suppression of viral load below detection limit does not completely reduce inflammation. As a result, long-term antiretrovirally treated patients still have an increased risk of death due to non-AIDS complications, which are typically associated with aging, for example, cardiovascular disease, osteoporosis, and end-organ failure [3]. Most notably, the prevalence of non-AIDS-associated malignancies is increasing, for example, Epstein-Barr virus-associated Hodgkin lymphoma and human papillomavirus-associated anal neoplasia [4].

Do HIV-1-infected subjects now just live long enough to experience their tumor? Or are these complications a result of the persistent immune activation? If this is the case, we need to think about adjunctive therapeutical approaches to limit the level of immune activation. One of the most proinflammatory messengers in the body is the type I interferons. Therefore, this review aims to link current models of IFN-alpha induction and suppression in human and simian models of lentiviral infections with the results of most recent clinical studies. The readers are also referred to three excellent reviews which summarize the findings about the HIV-1-induced immunopathogenesis [5–7].

## 2. Beyond HIV

“Complete” inhibition of viral replication—measured by the reduction of the viral load below detection limit—is obviously not sufficient to reverse the HIV-1-induced immunological damage in all patients. One reason may be that the peripheral viral load does not reliably reflect the situation in the lymphatic tissue [8]. Another explanation is that the HIV-1-associated chronic immune activation, which has recently come into the focus of scientific interest, plays a more important role than previously thought. Early studies in HIV-1-infected patients showed that the progression to AIDS was more strongly associated with levels of chronic immune activation than with viral loads [9–13]. Not only in humans, but also in the macaque model, it was shown that immune activation played a crucial role in lentiviral pathogenesis. The infection with simian immunodeficiency viruses (SIV) is usually apathogenic in the natural hosts like sooty mangabeys or African green monkeys [14, 15], whereas the infection of rhesus macaques with pathogenic SIV strains resembles the progressive course of human HIV-1 infection [16]. Pathogenic and apathogenic lentiviral infections are characterized by comparable viral loads in acute and chronic phases of the disease and a similar degree of CD4+ T cell destruction in cell culture [7]. In contrast, T-cell activation and programmed cell death as well as the secretion of proinflammatory cytokines are significantly enhanced in pathogenic infections, which progress with a decline in helper T cells and the occurrence of opportunistic infections [7].

## 3. Trigger of Immune Stimulation

An important stimulus for the chronic immune activation is the massive destruction of CD4+ T cells in the gastrointestinal tract, which has been observed in primary and chronic HIV-1 and SIV infections [17–22]. The resulting breach of the mucosal barrier supports the translocation of intestinal bacteria, which induces the production of proinflammatory cytokines, type I interferons and a systemic immune activation through the lipopolysaccharide (LPS) of gram-negative species, and other microbial constituents [23]. As an indicator of microbial translocation, circulating LPS levels are elevated in HIV-1-infected individuals [17, 23] and SIV-infected rhesus macaques [24]. Increased LPS levels in the plasma were correlated with chronic immune activation and were only partially reduced after administration of antiretroviral therapy [23].

Another potent trigger of immune stimulation is the type I interferons. In 1957, Alick Isaacs und Jean Lindenmann discovered a soluble substance, termed “interferon”, which interfered with the influenza virus infection in cell culture [25]. Meanwhile, a whole family of interferons and interferon-like molecules have been identified [26]. Amongst them are the strongly antiviral, antiproliferative, and immunomodulatory type I interferons, mostly comprising IFN-alpha and IFN-beta. In 1999, the plasmacytoid dendritic cells (PDC) were identified as major producers of these interferons in the blood [27, 28]. Although accounting for only 0.2–0.5% of peripheral blood mononuclear cells, PDC produce up to 1000-fold more IFN-alpha than any other cell in the body [29]. Armed with the endosomal Toll-like receptors (TLR) 7 and 9, PDC detect single-stranded RNA and CpG-like DNA molecules, respectively [30]. These cells drive a potent Th1 polarisation upon stimulation with respective bacterial and viral ligands, thus acting as “watchdogs” of the immune system [31, 32]. It should be considered that other cell types besides PDC also produce type I IFN, which may be important in particular on a tissue or host basis.

## 4. The Endosomal Enigma

High-titered infectious and noninfectious HIV-1 [33–38] and in particular HIV-1-infected cells [39, 40] induce a robust IFN-alpha production. The attachment of virions to PDC is crucially dependent on binding of the viral envelope protein gp120 to the CD4 receptor on PDC, supported by the finding that the degree of IFN-alpha induction is correlated with the affinity of the virus to CD4 [41]. In addition, the PDC-specific C-type lectin BDCA2 [42] and the mannose receptor [43] were reported to be involved in the attachment of HIV-1 to PDC. After binding, HIV-1 can interact with the coreceptors and infect the PDC [44], which has been observed for CCR5- and CXCR4-tropic viruses [38, 40, 45–47]. Productive infection can be enhanced by maturation of PDC via CD40 ligand, which reduces their capacity to produce type I interferons, or by neutralizing secreted interferons using respective antibodies [34, 44].

The induction of type I interferon production by HIV-1, however, is independent of coreceptor usage and fusion with the plasma membrane [33, 37, 40, 41]. Notably, the IFN-alpha induction could be blocked by drugs which interfere with the endosomal acidification, for example, chloroquine, bafilomycin, ammonium chloride, and chlorpromazine [33, 39, 40, 48]. Together with the colocalization of HIV-1 with the early endosomal antigen 1 in PDC [49], these findings suggest that virions are taken up into endosomal compartments of PDC. A key observation was that virions which were no longer able to package viral RNA were severely impaired in the IFN-alpha induction, indicating that viral nucleic acids are required for the activation of PDC [33]. This finding is consistent with the IFN-alpha induction by guanosin- and uridine-rich single-stranded RNA motifs derived from the 5′ untranslated region of HIV-1, which have been reported to interact with TLR7 and TLR8 [50]. Whether TLR9 also plays a role in this process is still controversially discussed. In this respect, we observed that the IFN-alpha induction by HIV-1-infected cells was blocked by lower concentrations of chloroquine than a synthetic TLR7 ligand [40]. When TLR-specific inhibitory oligonucleotides were used in the macaque model, a role for TLR9 in the induction of IFN-alpha production by SIV could not be excluded [51]. In a most recent study, however, TLR7 was silenced by siRNA in a plasmacytoid cell line; thereby, the authors could show that the majority of IFN-alpha production by HIV-1-infected cells is through TLR7 [39]. Moreover, when 293T cells, which do not express endosomal TLRs, were transfected with CD4 and CXCR4 and subsequently exposed to HIV-1-infected cells, an IFN-beta promoter was induced [39]. These findings provide first evidence that HIV-1 RNA may be recognized by cytosolic pattern recognition receptors, which induces a type I interferon production through interferon response factor (IRF) 3.

**Figure 1:**
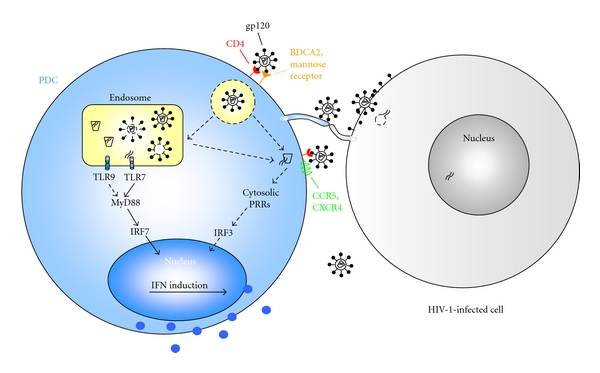
Proposed model of the type I interferon induction by HIV-1 and HIV-1-infected cells. Attachment to the CD4 receptor of plasmacytoid dendritic cells (PDC) triggers the HIV-1 uptake into an endosomal compartment. Subsequent acidification releases nucleic acids from lysed virions, preferentially recruits Toll-like receptor (TLR) 7, and activates the MyD88-dependent pathway. Translocation of the interferon response factor (IRF) 7 into the nucleus activates production of type I interferons (IFN). HIV-1 can also infect PDC via interaction with CD4 and the coreceptors. So far, it is unclear whether virions can escape from the endosomal compartment and initiate productive infection. Another pathway of type I interferon production was recently described in the absence of endosomal TLRs, namely, the recognition of HIV-1 RNA via cytosolic pattern recognition receptors (PRR) and IRF3 [39]. The role of this pathway for the IFN-alpha induction in PDC needs to be further elucidated.

Altogether, the data support a model (Figure 1), in which the IFN-alpha production is initiated by the endocytic uptake of virions into PDC. Nucleic acids are released upon endosomal acidification and preferentially interact with TLR7 to promote a signaling cascade via MyD88 and IRF7. The relevance of the TLR-independent recognition of HIV-1 in PDC still has to be analyzed.

## 5. The Vicious Circle of Type 1 Interferons

The induction of type I interferons has beneficial effects, as HIV-1-infected cells are driven into the programmed cell death [6], and viral replication is reduced by 1(−2)log [44, 52, 53]. However, the increased induction of IFN-alpha production by infectious and noninfectious virions is also detrimental, as addressed by several groups (reviewed in [54]). In this respect, it was shown by the group of Gene Shearer, NIH Bethesda, that the TNF-related apoptosis-inducing ligand (TRAIL) and its death receptor (DR) 5 was induced on CD4+ T lymphocytes in the peripheral blood and in secondary lymphatic tissue [36, 37, 55]. This mechanism leads to the apoptosis of uninfected CD4+ T cells, thereby contributing to the characteristic destruction of the lymph node architecture in advanced stages of HIV-1 infection. Moreover, the PDC-derived IFN-alpha induces the immunosuppressive enzyme indoleamine-2,3-dioxygenase (IDO), which leads to reduced CD4+ T-cell proliferation and T-cell dysfunction [56, 57] and enhanced activity of T-regulatory cells [58]. Concomitantly with these findings, a signature of increased IFN-alpha production, namely, the upregulation of IFN-responsive genes, is observed in peripheral cells and in the lymphatic tissue of HIV-1-infected individuals [36, 37, 55, 59, 60]. These data have been confirmed in primate lentiviral models, in which pathogenic SIV infections were associated with increased IFN-alpha and TRAIL levels [51, 61]. Further corroboration comes from studies of apathogenic SIV infections, in which the strong type I interferon response in acute infections was quickly downregulated, whereas persistent IFN-alpha production was observed during pathogenic SIV infection of rhesus macaques [62–65].

Is the immune stimulation reflected by an elevated level of circulating type I interferons in HIV-1-infected individuals? Older studies say so [66–68], but this has recently been questioned when similar IFN-alpha levels were measured in viremic and aviremic HIV-1-infected individuals and uninfected controls [69]. An explanation may be that elevated IFN-alpha levels in the periphery are primarily present in patients with end-stage disease. In countries with access to highly active antiretroviral therapy, however, these individuals have become rare. Another important aspect is that HIV-1-infected cells induce 10–100-fold more IFN-alpha compared to cell-free virions, as shown by the use of transwell chambers and gentle shaking of cocultures [39, 40]. These findings are consistent with an increased transfer of viruses by filopodial bridges [70]. Thus, the major release of IFN-alpha—and consequently its detrimental effects—appear to occur preferentially in the lymphatic tissue, where HIV-1-infected cells are in close contact with PDC, which enhances cell-to-cell transfer of HIV-1 [71]. In a recent *in vivo* study, PDC accumulated in the spleens of HIV-1-infected subjects [72]. Interestingly, IFN-alpha did not colocalize with PDC but other cells, for example, T and B cells, which may reflect uptake of the cytokine via IFN-alpha receptors [72]. Another important site is the mucosal tissue, to which PDC are recruited in pathogenic SIV infection of rhesus macaques [73].

The increased type I interferon production upon stimulation with HIV-1-infected cells in the lymphatic tissue is faced by a decreased IFN-alpha response of peripheral cells to TLR stimulation. On the one hand, this is due to reduced PDC counts in HIV-1 infection, which has been confirmed by many groups (reviewed in [5]). On the other hand, progressive disease comes along with functional PDC deficits, in particular, reduced IFN-alpha production upon stimulation with TLR7 and TLR9 agonists [74–78]. In early stages of HIV-1 infection (Fiebig V-VI), however, PDC retain the ability to respond to TLR7/8 stimulation [79]. Notably, numerical and functional PDC deficits are not completely restored by antiretroviral therapy [80, 81]. The ongoing innate immune defect may account for the increase of viral infections and associated tumors that are in principle susceptible to type I interferons. In this respect, it is intriguing to look at the spectrum of opportunistic infections in the immune reconstitution inflammatory syndrome, which occurs in about 20% of HIV-1-infected individuals on newly initiated antiretroviral therapy. Besides genital warts, there is a high frequency of genital herpes, molluscum contagiosum, and varicella-zoster virus [82], which are known or suspected to be TLR9 agonists [83]. Similarly striking is the increase of papillomavirus-associated anal neoplasia in HIV-1-infected patients despite antiretroviral therapy; these lesions are responsive to the TLR7 agonist imiquimod [4, 84].

**Figure 2:**
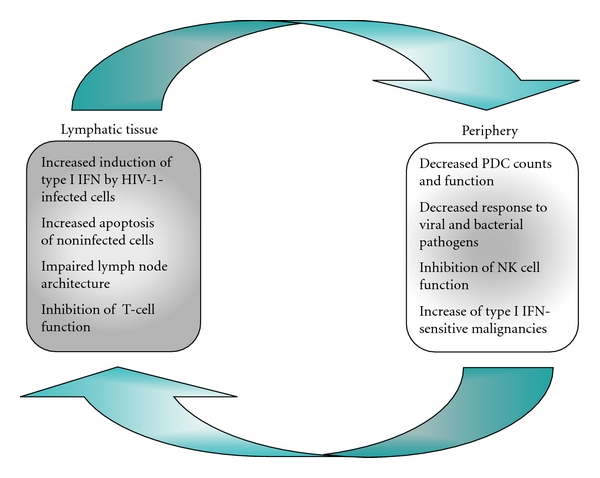
Vicious circle of type I interferon (IFN) induction in HIV-1 infection. The IFN-alpha induction is no longer balanced in HIV-1 infection. In the lymphatic tissue, plasmacytoid dendritic cells (PDC) are activated through direct cell-to-cell contact with HIV-1-infected cells, which creates an interferon-rich environment, promotes the apoptosis of uninfected T cells, inhibits T-cell functions, and destroys the lymph node architecture. In the periphery, reduced PDC counts and function result in an impaired innate immune response to bacterial and viral stimuli. Decreased natural killer (NK) cell functions may enhance the susceptibility to opportunistic infections and virus-induced tumor growth. The occurrence of pathogens in the periphery further causes PDC activation and depletion into lymphatic tissues.

How can the reduced responsiveness of PDC to TLR stimulation in the periphery be put together with the enhanced IFN-alpha release particularly in the lymphatic tissue? Tilton and colleagues provided an appealing explanation saying that PDC were preactivated via type I IFNs or virions *in vivo*, which prohibited de novo stimulation *in vitro* [60]. This has been questioned by a recent study, which showed prolonged and repeated IFN-alpha signaling in PDC exposed to HIV-1 due to an inability of endosomes to mature [49]. Another hypothesis may be that the HIV-1-induced immune stimulation somehow actively suppresses the induction of IFN-alpha production. As a result, PDC are no longer able to fulfill their purpose as “watchdogs” of the immune system: they now resemble “a dog that bites its tail.” A model for the ambiguous role of type I interferons in the immunopathogenesis of HIV-1 infection is proposed in Figure 2.

## 6. From Bench to Bedside

If one adopts the concept of chronic immune activation as a major factor in the HIV-1 progression, the therapeutic consequence would be to limit this immune stimulation. A proof of principle was provided by early studies using low-dose prednisolone, which significantly stabilized CD4+ T-cell counts in otherwise untreated HIV-1-infected patients [85]. These data were corroborated in individuals on HAART, although the effect on the CD4+ T cells was smaller [86]. In early studies by the group of Zagury, a total of 27 and 242 HIV-1-infected subjects were vaccinated against IFN-alpha-2b in a phase I/II study and a double-blind placebo-controlled phase II/III clinical trial, respectively [87, 88]. Although the immunogenicity of the vaccine was low, individuals who responded to vaccination had a lower rate of disease progression.

A different approach was used in recent studies, which focused on the effect of chloroquine on the HIV-1-related immune activation (summarized in Table 1). Chloroquine, which interferes with the endosomal acidification, is licensed for the prophylactic treatment of malaria. It is also widely used in the treatment of autoimmune disorders, for example, the rheumatoid arthritis, to curb the chronic immune activation. In cell culture, chloroquine inhibited the induction of IFN-alpha production by HIV-1 and HIV-1-infected cells, as outlined above. Chloroquine also decreased CD8+ T-cell activation and blocked two negative regulators of the T-cell response, IDO, and programmed death ligand 1 [89]. When mice were treated with chloroquine, the production of proinflammatory cytokines upon stimulation with LPS was reduced [90]. These data were recently supported and extended by two clinical trials with HIV-1-infected individuals. In the first study, chloroquine was administered to 13 HAART-naïve subjects for 2 months [91]. The patients showed immunological improvement, as the frequency of CD38+ HLA-DR+ CD8+ T cells, the proliferation of T cells, and circulating LPS levels were significantly reduced. The most recent study included a total of 20 HIV-1-infected subjects, who suffered from inadequate reconstitution of CD4+ T cells despite suppressive antiretroviral therapy [92]. These immunological nonresponders received chloroquine for six months in addition to HAART, which was well tolerated except for a skin rash in one case. Several immunological parameters improved as circulating LPS levels and activation of CD4+ T cells and monocytes, and the production of inflammatory cytokines (IL-6, TNF-alpha) was reduced, documenting the immunomodulatory effect of adjuvant chloroquine treatment. Of note, the percentage (however not the absolute count) of circulating CD4+ T cells and PDC increased, suggesting that sustained reduction of immune activation translates into clinical response. These preliminary data, however, should be interpreted with caution and need to be reproduced in larger cohorts of HIV-1-infected individuals. Such studies are in progress (ACTG A5258, http://clinicaltrials.gov/ct2/show/NCT00819390; CTN 246, http://www.hivnet.ubc.ca/clinical-studies/canadian-hiv-trials-database/ctn-246/).

## 7. Immune Modulation In Vivo

The endosome is an interesting target for immune modulation, because drugs acting at this compartment are not associated with broad immunosuppression, which is well known for high-dose corticoid treatment. In this respect, it is interesting that chloroquine was enriched in adenoid tissues, thus being targeted to the center of the HIV-1-induced immunopathogenesis [93]. It should be considered that chloroquine may aggravate the peripheral unresponsiveness of PDC to TLR stimulation, as the IFN-alpha induction by CpG oligodeoxynucleotides and synthetic TLR7 agonists can also be blocked by chloroquine. These findings may explain why chloroquine administration in otherwise untreated HIV-1-infected patients has not prevailed in the past 15 years. Thus, chloroquine appears to be promising only if used in combination with suppressive antiretroviral therapy. Long-term studies will show whether chloroquine will help to restore PDC counts and function in the periphery, which will hopefully also reduce the frequency of non-AIDS-associated tumors. It also needs to be investigated whether chloroquine suffices to revert all signs of chronic immune activation. It may well be possible that a few HIV-1-infected patients will profit from a more potent reduction of immune activation. Potential candidates are biologicals used for the treatment of autoimmune diseases, for example, IL-6 receptor antagonists, antibodies against TNF-alpha, or soluble TNF-alpha receptors.

## 8. Concluding Remarks

The concept of chronic immune activation in the immunopathogenesis of HIV-1 infection has opened the door for adjuvant immunomodulatory therapies. Future will tell whether the antiretroviral therapy and quality of life of HIV-1-infected individuals can further be improved. Continuous efforts of basic and applied sciences and pharmaceutical companies are required to stay on an upward track.

## Figures and Tables

**Table 1:** Effect of chloroquine on the HIV-1 and SIV-induced type I interferon production and/or subsequent immune activation *in vitro* and *in vivo*.

Study	Design	Species	Read-out
[33, 40]	*In vitro*	Human	Reduced IFN-alpha production

[51]	*In vitro*	Rhesus	Reduced IFN-alpha production

[89]	*In vitro*	Human	Reduced IFN-alpha production, reduced CD8+ T-cell activation (CD38), block of negative modulators of the T-cell response (IDO, PDL-1), reduced PDC activation and maturation

[91]	*In vivo*	Human	Reduced CD8+ cell activation (CD38+ HLA-DR+), reduced CD4+ and CD8+ T-cell proliferation (Ki-67)

[92]	*In vivo*	Human	Reduced CD4+ T-cell proliferation (Ki-67), reduced activation of CD8+ T cells (CD38+ HLA-DR+) and monocytes (CD69), reduced plasma LPS levels, reduced proinflammatory cytokines (IL-6, TNF-alpha), increase in CD4+ T-cell percentages
